# Brusatol

**DOI:** 10.1107/S1600536812018582

**Published:** 2012-05-02

**Authors:** Shu-Zhi Hu, Lu Jin, Tong Yu, Hai-Yan Tian, Ren-Wang Jiang

**Affiliations:** a Guangdong Province Key Laboratory of Pharmacodynamic Constituents of, Traditional Chinese Medicine and New Drugs Research, Institute of Traditional Chinese Medicine and Natural Products, Jinan University, Guangzhou 510632, People’s Republic of China

## Abstract

The title compound, C_26_H_32_O_11_, is composed of an α,β-unsaturated cyclo­hexa­none ring (*A*), two cyclo­hexane rings (*B* and *C*), a six-membered lactone ring (*D*) and tetra­hydro­furan ring (*E*). Ring *A* exists in a half-chair conformation with a C atom displaced by 0.679 (2) Å from the mean plane through the remaining five atoms. Ring *B* exists in a normal chair conformation. Both rings *C* and *D* exist in a twisted-chair conformation due to the O-atom bridge and the carbonyl group in rings *C* and *D*, respectively. Ring *E* shows an envelope conformation with a C atom displaced by 0.761 (1) Å from the mean plane through the remaining five atoms. The ring junctions are *A*/*B trans*, *B*/*C trans*, *C*/*D cis* and *D*/*E cis*. An intra­molecular O—H⋯O hydrogen bond occurs. In the crystal, O—H⋯O hydrogen bonds involving the hy­droxy, lactone and ester groups and C—H⋯O inter­actions are observed.

## Related literature

For the isolation of brusatol, see: Sim *et al.* (1968 ▶); Kim *et al.* (2004 ▶). For its anti­cancer activity, see: Zhao *et al.* (2011 ▶) and for its anti­viral activity, see: Yan *et al.* (2010 ▶). For the enhancement of the efficacy for chemotherapy, see: Ren *et al.* (2011 ▶). For the crystal structure of bruceine A, see: Feng *et al.* 2010 ▶. For the absolute configuration of simalikalactone D, see: Moher *et al.* (1992 ▶).
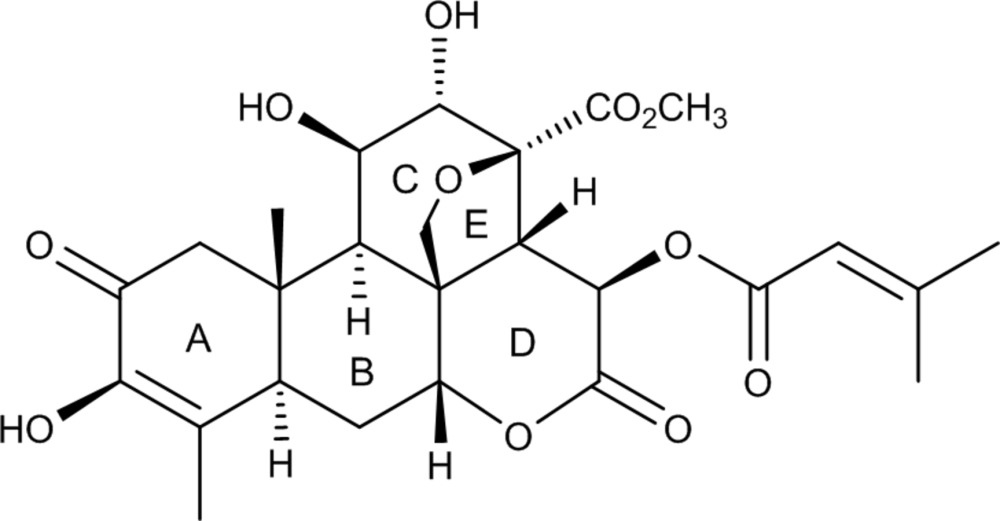


## Experimental

### 

#### Crystal data

C_26_H_32_O_11_
*M* *_r_* = 520.52
Orthorhombic, 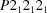

*a* = 6.7162 (1) Å
*b* = 13.6796 (2) Å
*c* = 25.9859 (5) Å
*V* = 2387.45 (7) Å^3^
*Z* = 4
Cu *K*α radiation
μ = 0.96 mm^−1^
*T* = 288 K
0.44 × 0.15 × 0.11 mm

#### Data collection

Oxford Gemini S Ultra Sapphire CCD diffractometer
Absorption correction: multi-scan (*CrysAlis PRO*; Agilent, 2011 ▶) *T*_min_ = 0.748, *T*_max_ = 1.000
4961 measured reflections
3380 independent reflections
3182 reflections with *I* > 2σ(*I*)
*R*_int_ = 0.020

#### Refinement

*R*[*F* ^2^ > 2σ(*F* ^2^)] = 0.032
*wR*(*F* ^2^) = 0.085
*S* = 1.03
3380 reflections
338 parameters
H-atom parameters constrained
Δρ_max_ = 0.27 e Å^−3^
Δρ_min_ = −0.18 e Å^−3^
Absolute structure: Flack (1983 ▶), 1167 Friedel pairs
Flack parameter: −0.07 (19)

### 

Data collection: *CrysAlis PRO* (Agilent, 2011 ▶); cell refinement: *CrysAlis PRO*; data reduction: *CrysAlis PRO*; program(s) used to solve structure: *SHELXTL* (Sheldrick, 2008 ▶); program(s) used to refine structure: *SHELXTL*; molecular graphics: *XP* in *SHELXTL*; software used to prepare material for publication: *SHELXTL*.

## Supplementary Material

Crystal structure: contains datablock(s) I, global. DOI: 10.1107/S1600536812018582/vm2168sup1.cif

Structure factors: contains datablock(s) I. DOI: 10.1107/S1600536812018582/vm2168Isup2.hkl

Additional supplementary materials:  crystallographic information; 3D view; checkCIF report

## Figures and Tables

**Table 1:** Hydrogen-bond geometry (Å, °)

*D*—H⋯*A*	*D*—H	H⋯*A*	*D*⋯*A*	*D*—H⋯*A*
O2—H2*A*⋯O1	0.82	2.17	2.629 (3)	116
O3—H3*A*⋯O11^i^	0.82	2.09	2.911 (2)	173
O4—H4*A*⋯O9^ii^	0.82	2.41	3.180 (2)	157
O4—H4*A*⋯O8^ii^	0.82	2.33	3.066 (2)	149
C11—H11*A*⋯O9^ii^	0.98	2.54	3.368 (4)	142
C5′—H5′*B*⋯O1^iii^	0.96	2.76	3.650 (3)	155

Symmetry codes: (i) 
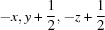
; (ii) 
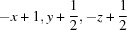
; (iii) 
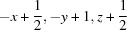
.

**Fig. 1.**
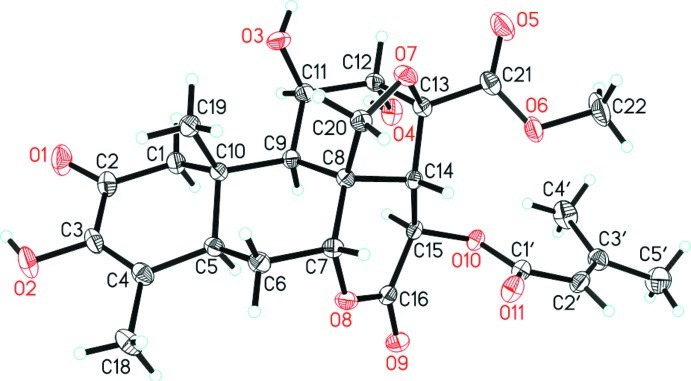
The molecular structure of the title compound showing 30% probability displacement ellipsoids.

**Fig. 2.**
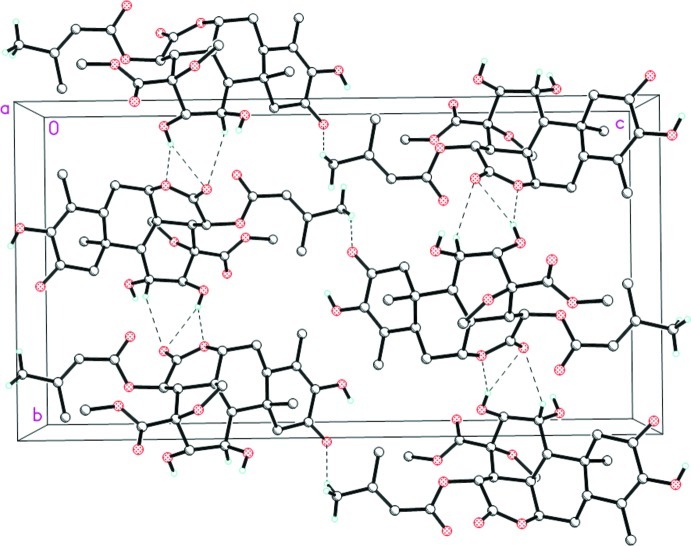
The packing diagram viewed down the a axis. The dashed lines represent intermolecular O—H···O hydrogen bonds and C—H···O short contacts. Selected H-atoms highlighting the hydrogen bondings and short contacts are shown.

## supplementary crystallographic information

### Comment

The title compound brusatol is a natural product originally isolated from the seeds of Brucea sumatrana (Sim *et al.*, 1968). It was also isolated from the chinese herbal medicine Brucea javanica (Kim *et al.*, 2004). Brusatol was found to show potent anticancer activity (Zhao *et al.*, 2011) and antiviral activity against the tobaccomosaic virus (Yan *et al.*, 2010). Furthermore, it was reported that brusatol could effectively enhance the efficacy of chemotherapy by inhibiting the Nrf2-mediated defense mechanism (Ren *et al.*, 2011). The crystal strcuture of bruceine A, an analogue of brusatol, was reported recently (Feng *et al.*, 2010); however, no detailed structural information was provided. We report herein the three-dimensional structure of the title compound.

Brusatol consists of an α,β-unsaturated cyclohexanone ring (A), two cyclohexane rings (B and C), a six-membered lactone ring (D) and tetrahydrofuran ring (E). Ring A exists in a half chair conformation with C10 displaced by 0.679 (2) Å from the least squares plane through the remaining five atoms (C1, C2, C3, C4 and C5). Ring B exists in a normal chair conformation. Both rings C and D exist in a twisted chair conformation due to the oxygen bridge and carbonyl group in rings C and D, respectively. Ring E shows an envelop conformation with C14 displaced by 0.761 (1) Å from the least squares plane through the remaining four atoms (C8, C13, C20 and O7). The planes through rings A and E are roughly perpendicular to each other with a dihedral angle of 86.15 (9)°. There are two side chains at C13 and C15. The planes through the two ester groups in the side chains make a dihedral angle of 62.36 (10). The ring junctures are A/B *trans*, B/C *trans*, C/D *cis* and D/E *cis*. The absolute configuration determined for simalikalactone D (Moher *et al.*, 1992), a similar quassinoid, was invoked, giving the assignments of the chiral centres in the molecule as shown in Fig. 1.

Intermolecular O–H···O hydrogen bonds (Table 1) between the hydroxyl groups at C11 and the ester group at C1' [O3···O11^i ^ = 2.911 (2) Å, symmetry code: (i) -*x*, 0.5 + *y*, 0.5 - *z*], between the hydroxyl group at C12 and the lactone group at C16 [O4···O8^ii^ = 3.066 (2) Å and O4···O9^ii^ = 3.180 (2) Å, symmetry code: (ii) 1 - *x*, 0.5 + *y*, 0.5 - *z*], and short C–H···O contacts between the methine group at C11 and the lactone group at C16 [C11···O9^ii^ = 3.368 (4) Å] link adjacent molecules into chains along the *b*-axis. Adjacent chains are further linked by weak C–H···O interactions between the terminal methyl group and the ketone group at C2 [C5'···O1^iii^ = 3.650 (3) Å, symmetry code: (iii) 0.5 - *x*, 1 - *y*, 0.5 + *z*] into a three-dimensional network (Fig. 2).

### Experimental

Dried seeds of brucea javanica (10 kg) were milled and extracted with 95% ethanol at room temperature and the extracted solution were concentrated to afford a syrup. The crude syrup was suspended in distilled water and partitioned with petroleum ether, ethyl acetate and n-butanol, successively. The ethyl acetate extract (82 g) was dissolved in warm methanol. After cooling, the raw brusatol precipitated as white powder. Then the powder (100 mg) was subjected to reverse phase HPLC to afford pure brusatol (83 mg). Colorless needles of the title compound were crystallized directly from the HPLC eluate acetonitrile: water 45:55.

### Refinement

The C-bound H atoms were positioned geometrically and were included in the refinement in the riding-model approximation, with C—H = 0.96 Å (CH_3_) and *U*_iso_(H) = 1.5*U*_eq_(C); 0.97 Å (CH_2_) and *U*_iso_(H) = 1.2*U*_eq_(C); 0.98 Å (CH) and *U*_iso_(H) = 1.2*U*_eq_(C); O—H = 0.82 Å and *U*_iso_(H) = 1.5*U*_eq_(O). The absolute configuration can be unambiguously assigned with reference to the known configuration of the closed related compound simalikalactone D. The Flack parameter was refined to -0.07 (19).

### Crystal data

**Table:**

C_26_H_32_O_11_	*D*_x_ = 1.448 Mg m^−^^3^
*M**_r_* = 520.52	Cu *K*α radiation, λ = 1.54184 Å
Orthorhombic, *P*2_1_2_1_2_1_	Cell parameters from 2860 reflections
*a* = 6.7162 (1) Å	θ = 3.2–62.6°
*b* = 13.6796 (2) Å	µ = 0.96 mm^−^^1^
*c* = 25.9859 (5) Å	*T* = 288 K
*V* = 2387.45 (7) Å^3^	Needle, colourless
*Z* = 4	0.44 × 0.15 × 0.11 mm
*F*(000) = 1104	

### Data collection

**Table:**

Oxford Gemini S Ultra Sapphire CCD diffractometer	3380 independent reflections
Radiation source: fine-focus sealed tube	3182 reflections with *I* > 2σ(*I*)
Mirror monochromator	*R*_int_ = 0.020
ω scan	θ_max_ = 62.6°, θ_min_ = 3.2°
Absorption correction: multi-scan (*CrysAlis PRO*; Agilent, 2011)	*h* = −7→6
*T*_min_ = 0.748, *T*_max_ = 1.000	*k* = −13→15
4961 measured reflections	*l* = −29→29

### Refinement

**Table:**

Refinement on *F*^2^	Hydrogen site location: inferred from neighbouring sites
Least-squares matrix: full	H-atom parameters constrained
*R*[*F*^2^ > 2σ(*F*^2^)] = 0.032	*w* = 1/[σ^2^(*F*_o_^2^) + (0.0463*P*)^2^ + 0.5393*P*] where *P* = (*F*_o_^2^ + 2*F*_c_^2^)/3
*wR*(*F*^2^) = 0.085	(Δ/σ)_max_ = 0.001
*S* = 1.03	Δρ_max_ = 0.27 e Å^−^^3^
3380 reflections	Δρ_min_ = −0.18 e Å^−^^3^
338 parameters	Extinction correction: *SHELXTL* (Sheldrick, 2008), Fc^*^=kFc[1+0.001xFc^2^λ^3^/sin(2θ)]^-1/4^
0 restraints	Extinction coefficient: 0.0070 (4)
Primary atom site location: structure-invariant direct methods	Absolute structure: Flack (1983), 1167 Friedel pairs
Secondary atom site location: difference Fourier map	Flack parameter: −0.07 (19)

### Special details

**Table:**

Geometry. All e.s.d.'s (except the e.s.d. in the dihedral angle between two l.s. planes) are estimated using the full covariance matrix. The cell e.s.d.'s are taken into account individually in the estimation of e.s.d.'s in distances, angles and torsion angles; correlations between e.s.d.'s in cell parameters are only used when they are defined by crystal symmetry. An approximate (isotropic) treatment of cell e.s.d.'s is used for estimating e.s.d.'s involving l.s. planes.
Refinement. Refinement of *F*^2^ against ALL reflections. The weighted *R*-factor *wR* and goodness of fit *S* are based on *F*^2^, conventional *R*-factors *R* are based on *F*, with *F* set to zero for negative *F*^2^. The threshold expression of *F*^2^ > σ(*F*^2^) is used only for calculating *R*-factors(gt) *etc*. and is not relevant to the choice of reflections for refinement. *R*-factors based on *F*^2^ are statistically about twice as large as those based on *F*, and *R*- factors based on ALL data will be even larger.

### Fractional atomic coordinates and isotropic or equivalent isotropic displacement parameters (Å^2^)

**Table:**

	*x*	*y*	*z*	*U*_iso_*/*U*_eq_	
O1	0.6704 (3)	0.54720 (14)	0.02225 (7)	0.0561 (6)	
O2	0.7863 (3)	0.37039 (16)	−0.00367 (7)	0.0551 (5)	
H2A	0.7975	0.4234	−0.0181	0.094 (15)*	
O3	0.0404 (3)	0.54519 (13)	0.14364 (6)	0.0441 (5)	
H3A	−0.0296	0.5919	0.1516	0.080 (12)*	
O4	0.2361 (3)	0.56662 (12)	0.27424 (6)	0.0411 (4)	
H4A	0.2887	0.6182	0.2656	0.069 (11)*	
O5	−0.2473 (3)	0.51540 (14)	0.30530 (8)	0.0653 (6)	
O6	−0.0670 (2)	0.39701 (16)	0.34093 (6)	0.0505 (5)	
O7	−0.1327 (2)	0.40828 (12)	0.21639 (6)	0.0342 (4)	
O8	0.4711 (2)	0.23809 (11)	0.21922 (6)	0.0349 (4)	
O9	0.6417 (3)	0.25571 (13)	0.28957 (6)	0.0424 (4)	
O10	0.3429 (2)	0.35608 (10)	0.33806 (5)	0.0281 (3)	
O11	0.2397 (3)	0.20076 (11)	0.33187 (6)	0.0389 (4)	
C1	0.5140 (4)	0.50092 (16)	0.10202 (8)	0.0332 (5)	
H1A	0.4364	0.5605	0.0990	0.040*	
H1B	0.6112	0.5104	0.1292	0.040*	
C2	0.6203 (4)	0.48223 (18)	0.05223 (9)	0.0377 (6)	
C3	0.6762 (4)	0.38148 (18)	0.04044 (9)	0.0370 (6)	
C4	0.6333 (4)	0.30630 (17)	0.07122 (8)	0.0333 (5)	
C5	0.5044 (3)	0.32145 (15)	0.11842 (8)	0.0273 (5)	
H5A	0.5971	0.3306	0.1471	0.033*	
C6	0.3745 (4)	0.23359 (15)	0.13322 (8)	0.0311 (5)	
H6A	0.2654	0.2271	0.1090	0.037*	
H6B	0.4532	0.1742	0.1319	0.037*	
C7	0.2931 (3)	0.24785 (15)	0.18675 (8)	0.0276 (5)	
H7A	0.2018	0.1940	0.1946	0.033*	
C8	0.1859 (3)	0.34436 (15)	0.19626 (8)	0.0237 (5)	
C9	0.2988 (3)	0.43333 (15)	0.17275 (8)	0.0247 (5)	
H9A	0.4203	0.4388	0.1934	0.030*	
C10	0.3752 (3)	0.41578 (15)	0.11664 (8)	0.0256 (5)	
C11	0.1883 (4)	0.53042 (16)	0.18209 (8)	0.0309 (5)	
H11A	0.2859	0.5833	0.1788	0.037*	
C12	0.0966 (3)	0.53777 (15)	0.23619 (8)	0.0293 (5)	
H12A	−0.0104	0.5864	0.2352	0.035*	
C13	0.0083 (3)	0.44045 (16)	0.25471 (8)	0.0293 (5)	
C14	0.1578 (3)	0.35654 (15)	0.25474 (8)	0.0236 (4)	
H14A	0.0874	0.2983	0.2671	0.028*	
C15	0.3574 (3)	0.35998 (15)	0.28264 (7)	0.0250 (4)	
H15A	0.4201	0.4225	0.2738	0.030*	
C16	0.4981 (4)	0.27969 (16)	0.26507 (8)	0.0299 (5)	
C18	0.7205 (4)	0.2074 (2)	0.06165 (10)	0.0464 (7)	
H18A	0.7984	0.2089	0.0307	0.070*	
H18B	0.6152	0.1605	0.0580	0.070*	
H18C	0.8038	0.1893	0.0901	0.070*	
C19	0.2138 (4)	0.40703 (18)	0.07452 (8)	0.0340 (5)	
H19A	0.2763	0.3965	0.0418	0.051*	
H19B	0.1371	0.4662	0.0734	0.051*	
H19C	0.1278	0.3530	0.0823	0.051*	
C20	−0.0349 (3)	0.34250 (16)	0.18165 (8)	0.0302 (5)	
H20A	−0.0530	0.3637	0.1463	0.036*	
H20B	−0.0884	0.2770	0.1852	0.036*	
C21	−0.1136 (4)	0.45655 (17)	0.30335 (9)	0.0355 (6)	
C22	−0.1880 (5)	0.4031 (3)	0.38682 (10)	0.0779 (11)	
H22A	−0.1412	0.3565	0.4117	0.117*	
H22B	−0.3240	0.3889	0.3783	0.117*	
H22C	−0.1790	0.4677	0.4010	0.117*	
C1'	0.2828 (3)	0.27079 (16)	0.35871 (8)	0.0308 (5)	
C2'	0.2793 (4)	0.26745 (17)	0.41472 (8)	0.0365 (6)	
H2'A	0.2646	0.2051	0.4284	0.044*	
C3'	0.2934 (4)	0.33837 (18)	0.44969 (9)	0.0384 (6)	
C4'	0.3105 (5)	0.44546 (19)	0.43916 (10)	0.0527 (7)	
H4'A	0.3144	0.4562	0.4027	0.079*	
H4'B	0.4305	0.4701	0.4545	0.079*	
H4'C	0.1977	0.4788	0.4536	0.079*	
C5'	0.2881 (5)	0.3129 (2)	0.50613 (9)	0.0623 (9)	
H5'A	0.2802	0.2432	0.5100	0.093*	
H5'B	0.1739	0.3427	0.5218	0.093*	
H5'C	0.4070	0.3365	0.5225	0.093*	

### Atomic displacement parameters (Å^2^)

**Table:**

	*U*^11^	*U*^22^	*U*^33^	*U*^12^	*U*^13^	*U*^23^
O1	0.0727 (14)	0.0511 (11)	0.0444 (10)	−0.0068 (11)	0.0227 (11)	0.0081 (9)
O2	0.0584 (12)	0.0674 (13)	0.0396 (10)	−0.0005 (11)	0.0244 (9)	−0.0064 (10)
O3	0.0530 (11)	0.0436 (10)	0.0356 (9)	0.0246 (9)	−0.0004 (8)	0.0049 (7)
O4	0.0518 (10)	0.0336 (9)	0.0378 (9)	−0.0135 (9)	0.0035 (8)	−0.0034 (7)
O5	0.0682 (14)	0.0472 (11)	0.0805 (14)	0.0163 (11)	0.0418 (12)	0.0069 (10)
O6	0.0322 (9)	0.0896 (14)	0.0296 (8)	0.0093 (10)	0.0090 (8)	0.0096 (9)
O7	0.0231 (7)	0.0424 (9)	0.0372 (8)	0.0033 (7)	−0.0018 (7)	−0.0045 (7)
O8	0.0376 (9)	0.0365 (8)	0.0308 (8)	0.0146 (8)	−0.0027 (8)	−0.0025 (7)
O9	0.0359 (9)	0.0523 (10)	0.0388 (9)	0.0117 (8)	−0.0061 (8)	0.0073 (8)
O10	0.0336 (8)	0.0287 (8)	0.0219 (7)	−0.0042 (7)	−0.0026 (7)	0.0022 (6)
O11	0.0555 (11)	0.0297 (8)	0.0314 (8)	−0.0101 (8)	−0.0033 (8)	−0.0006 (7)
C1	0.0356 (12)	0.0338 (12)	0.0301 (11)	−0.0011 (11)	0.0055 (10)	−0.0005 (9)
C2	0.0378 (13)	0.0440 (14)	0.0311 (12)	−0.0058 (12)	0.0058 (11)	0.0008 (11)
C3	0.0319 (12)	0.0496 (15)	0.0295 (11)	0.0001 (11)	0.0092 (10)	−0.0069 (11)
C4	0.0285 (11)	0.0406 (13)	0.0307 (11)	0.0020 (11)	−0.0014 (11)	−0.0105 (11)
C5	0.0256 (11)	0.0306 (11)	0.0259 (10)	0.0027 (10)	−0.0019 (10)	−0.0041 (9)
C6	0.0355 (12)	0.0257 (11)	0.0321 (11)	0.0020 (11)	0.0011 (11)	−0.0056 (9)
C7	0.0275 (11)	0.0249 (10)	0.0303 (11)	−0.0003 (9)	−0.0016 (10)	−0.0014 (9)
C8	0.0234 (11)	0.0241 (11)	0.0235 (10)	−0.0005 (9)	0.0000 (9)	−0.0037 (8)
C9	0.0257 (10)	0.0254 (10)	0.0230 (10)	−0.0007 (9)	−0.0004 (9)	−0.0014 (8)
C10	0.0239 (10)	0.0283 (11)	0.0248 (11)	0.0021 (10)	0.0012 (9)	−0.0004 (9)
C11	0.0362 (12)	0.0241 (10)	0.0323 (11)	0.0037 (10)	0.0055 (10)	0.0037 (9)
C12	0.0328 (12)	0.0235 (11)	0.0316 (11)	0.0052 (10)	0.0058 (10)	0.0007 (9)
C13	0.0266 (10)	0.0305 (12)	0.0307 (11)	−0.0006 (10)	0.0038 (10)	−0.0009 (9)
C14	0.0246 (10)	0.0229 (10)	0.0234 (10)	−0.0025 (9)	0.0009 (9)	0.0010 (8)
C15	0.0271 (10)	0.0270 (10)	0.0209 (9)	−0.0019 (9)	−0.0010 (9)	0.0042 (9)
C16	0.0308 (12)	0.0301 (12)	0.0289 (11)	0.0009 (10)	0.0015 (10)	0.0068 (9)
C18	0.0416 (14)	0.0490 (15)	0.0487 (15)	0.0056 (13)	0.0082 (13)	−0.0169 (12)
C19	0.0337 (12)	0.0423 (13)	0.0260 (11)	0.0037 (12)	−0.0039 (10)	−0.0007 (10)
C20	0.0257 (11)	0.0352 (12)	0.0299 (11)	−0.0013 (10)	−0.0024 (10)	−0.0015 (9)
C21	0.0325 (12)	0.0319 (12)	0.0421 (13)	−0.0048 (11)	0.0101 (11)	−0.0058 (11)
C22	0.0446 (17)	0.157 (4)	0.0324 (15)	0.009 (2)	0.0143 (13)	0.0076 (19)
C1'	0.0333 (12)	0.0300 (12)	0.0291 (11)	−0.0013 (11)	0.0003 (10)	0.0034 (10)
C2'	0.0463 (14)	0.0349 (12)	0.0284 (11)	0.0027 (12)	−0.0004 (11)	0.0069 (10)
C3'	0.0368 (13)	0.0484 (14)	0.0299 (12)	0.0042 (12)	−0.0002 (11)	−0.0020 (11)
C4'	0.0618 (18)	0.0455 (15)	0.0508 (16)	−0.0112 (15)	0.0082 (15)	−0.0182 (13)
C5'	0.081 (2)	0.077 (2)	0.0292 (13)	0.019 (2)	−0.0019 (15)	−0.0030 (14)

### Geometric parameters (Å, º)

**Table:**

O1—C2	1.229 (3)	C9—C11	1.541 (3)
O2—C3	1.372 (3)	C9—C10	1.564 (3)
O2—H2A	0.8200	C9—H9A	0.9800
O3—C11	1.423 (3)	C10—C19	1.545 (3)
O3—H3A	0.8200	C11—C12	1.538 (3)
O4—C12	1.418 (3)	C11—H11A	0.9800
O4—H4A	0.8200	C12—C13	1.535 (3)
O5—C21	1.207 (3)	C12—H12A	0.9800
O6—C21	1.310 (3)	C13—C21	1.522 (3)
O6—C22	1.445 (3)	C13—C14	1.525 (3)
O7—C20	1.434 (3)	C14—C15	1.525 (3)
O7—C13	1.443 (3)	C14—H14A	0.9800
O8—C16	1.333 (3)	C15—C16	1.519 (3)
O8—C7	1.469 (3)	C15—H15A	0.9800
O9—C16	1.201 (3)	C18—H18A	0.9600
O10—C1'	1.346 (3)	C18—H18B	0.9600
O10—C15	1.444 (2)	C18—H18C	0.9600
O11—C1'	1.220 (3)	C19—H19A	0.9600
C1—C2	1.500 (3)	C19—H19B	0.9600
C1—C10	1.539 (3)	C19—H19C	0.9600
C1—H1A	0.9700	C20—H20A	0.9700
C1—H1B	0.9700	C20—H20B	0.9700
C2—C3	1.461 (3)	C22—H22A	0.9600
C3—C4	1.334 (3)	C22—H22B	0.9600
C4—C18	1.495 (3)	C22—H22C	0.9600
C4—C5	1.516 (3)	C1'—C2'	1.456 (3)
C5—C6	1.534 (3)	C2'—C3'	1.333 (3)
C5—C10	1.556 (3)	C2'—H2'A	0.9300
C5—H5A	0.9800	C3'—C4'	1.495 (4)
C6—C7	1.507 (3)	C3'—C5'	1.508 (3)
C6—H6A	0.9700	C4'—H4'A	0.9600
C6—H6B	0.9700	C4'—H4'B	0.9600
C7—C8	1.524 (3)	C4'—H4'C	0.9600
C7—H7A	0.9800	C5'—H5'A	0.9600
C8—C20	1.531 (3)	C5'—H5'B	0.9600
C8—C14	1.540 (3)	C5'—H5'C	0.9600
C8—C9	1.559 (3)		
			
C3—O2—H2A	109.5	O7—C13—C21	105.32 (18)
C11—O3—H3A	109.5	O7—C13—C14	101.70 (16)
C12—O4—H4A	109.5	C21—C13—C14	117.56 (19)
C21—O6—C22	116.4 (2)	O7—C13—C12	107.57 (17)
C20—O7—C13	108.99 (16)	C21—C13—C12	110.05 (18)
C16—O8—C7	125.83 (17)	C14—C13—C12	113.48 (18)
C1'—O10—C15	116.72 (16)	C15—C14—C13	123.77 (18)
C2—C1—C10	111.86 (18)	C15—C14—C8	111.37 (17)
C2—C1—H1A	109.2	C13—C14—C8	99.31 (16)
C10—C1—H1A	109.2	C15—C14—H14A	107.1
C2—C1—H1B	109.2	C13—C14—H14A	107.1
C10—C1—H1B	109.2	C8—C14—H14A	107.1
H1A—C1—H1B	107.9	O10—C15—C16	108.35 (16)
O1—C2—C3	118.6 (2)	O10—C15—C14	114.43 (17)
O1—C2—C1	123.6 (2)	C16—C15—C14	112.44 (17)
C3—C2—C1	117.6 (2)	O10—C15—H15A	107.1
C4—C3—O2	122.1 (2)	C16—C15—H15A	107.1
C4—C3—C2	123.1 (2)	C14—C15—H15A	107.1
O2—C3—C2	114.7 (2)	O9—C16—O8	117.8 (2)
C3—C4—C18	120.9 (2)	O9—C16—C15	122.5 (2)
C3—C4—C5	120.2 (2)	O8—C16—C15	119.52 (18)
C18—C4—C5	118.8 (2)	C4—C18—H18A	109.5
C4—C5—C6	114.88 (17)	C4—C18—H18B	109.5
C4—C5—C10	114.09 (17)	H18A—C18—H18B	109.5
C6—C5—C10	109.88 (17)	C4—C18—H18C	109.5
C4—C5—H5A	105.7	H18A—C18—H18C	109.5
C6—C5—H5A	105.7	H18B—C18—H18C	109.5
C10—C5—H5A	105.7	C10—C19—H19A	109.5
C7—C6—C5	109.64 (17)	C10—C19—H19B	109.5
C7—C6—H6A	109.7	H19A—C19—H19B	109.5
C5—C6—H6A	109.7	C10—C19—H19C	109.5
C7—C6—H6B	109.7	H19A—C19—H19C	109.5
C5—C6—H6B	109.7	H19B—C19—H19C	109.5
H6A—C6—H6B	108.2	O7—C20—C8	106.08 (17)
O8—C7—C6	102.92 (17)	O7—C20—H20A	110.5
O8—C7—C8	111.71 (16)	C8—C20—H20A	110.5
C6—C7—C8	115.66 (18)	O7—C20—H20B	110.5
O8—C7—H7A	108.8	C8—C20—H20B	110.5
C6—C7—H7A	108.8	H20A—C20—H20B	108.7
C8—C7—H7A	108.8	O5—C21—O6	124.2 (2)
C7—C8—C20	113.77 (18)	O5—C21—C13	122.1 (2)
C7—C8—C14	108.16 (17)	O6—C21—C13	113.6 (2)
C20—C8—C14	97.32 (17)	O6—C22—H22A	109.5
C7—C8—C9	112.53 (17)	O6—C22—H22B	109.5
C20—C8—C9	112.77 (17)	H22A—C22—H22B	109.5
C14—C8—C9	111.21 (16)	O6—C22—H22C	109.5
C11—C9—C8	112.15 (17)	H22A—C22—H22C	109.5
C11—C9—C10	115.92 (17)	H22B—C22—H22C	109.5
C8—C9—C10	113.89 (16)	O11—C1'—O10	121.61 (19)
C11—C9—H9A	104.5	O11—C1'—C2'	122.9 (2)
C8—C9—H9A	104.5	O10—C1'—C2'	115.5 (2)
C10—C9—H9A	104.5	C3'—C2'—C1'	131.1 (2)
C1—C10—C19	107.97 (17)	C3'—C2'—H2'A	114.4
C1—C10—C5	107.28 (17)	C1'—C2'—H2'A	114.4
C19—C10—C5	110.36 (17)	C2'—C3'—C4'	126.4 (2)
C1—C10—C9	108.21 (17)	C2'—C3'—C5'	119.6 (2)
C19—C10—C9	116.26 (18)	C4'—C3'—C5'	114.0 (2)
C5—C10—C9	106.41 (16)	C3'—C4'—H4'A	109.5
O3—C11—C12	110.70 (18)	C3'—C4'—H4'B	109.5
O3—C11—C9	110.37 (17)	H4'A—C4'—H4'B	109.5
C12—C11—C9	113.15 (17)	C3'—C4'—H4'C	109.5
O3—C11—H11A	107.5	H4'A—C4'—H4'C	109.5
C12—C11—H11A	107.5	H4'B—C4'—H4'C	109.5
C9—C11—H11A	107.5	C3'—C5'—H5'A	109.5
O4—C12—C13	106.15 (17)	C3'—C5'—H5'B	109.5
O4—C12—C11	113.04 (18)	H5'A—C5'—H5'B	109.5
C13—C12—C11	112.62 (17)	C3'—C5'—H5'C	109.5
O4—C12—H12A	108.3	H5'A—C5'—H5'C	109.5
C13—C12—H12A	108.3	H5'B—C5'—H5'C	109.5
C11—C12—H12A	108.3		
			
C10—C1—C2—O1	149.1 (2)	O3—C11—C12—C13	85.9 (2)
C10—C1—C2—C3	−34.3 (3)	C9—C11—C12—C13	−38.6 (3)
O1—C2—C3—C4	178.2 (2)	C20—O7—C13—C21	−148.07 (18)
C1—C2—C3—C4	1.5 (4)	C20—O7—C13—C14	−24.9 (2)
O1—C2—C3—O2	1.5 (4)	C20—O7—C13—C12	94.6 (2)
C1—C2—C3—O2	−175.2 (2)	O4—C12—C13—O7	−179.23 (16)
O2—C3—C4—C18	6.0 (4)	C11—C12—C13—O7	−55.0 (2)
C2—C3—C4—C18	−170.5 (2)	O4—C12—C13—C21	66.5 (2)
O2—C3—C4—C5	−177.6 (2)	C11—C12—C13—C21	−169.28 (19)
C2—C3—C4—C5	6.0 (4)	O4—C12—C13—C14	−67.5 (2)
C3—C4—C5—C6	148.1 (2)	C11—C12—C13—C14	56.6 (2)
C18—C4—C5—C6	−35.4 (3)	O7—C13—C14—C15	170.06 (18)
C3—C4—C5—C10	19.9 (3)	C21—C13—C14—C15	−75.6 (3)
C18—C4—C5—C10	−163.6 (2)	C12—C13—C14—C15	54.8 (3)
C4—C5—C6—C7	166.34 (18)	O7—C13—C14—C8	46.42 (19)
C10—C5—C6—C7	−63.4 (2)	C21—C13—C14—C8	160.78 (19)
C16—O8—C7—C6	152.39 (19)	C12—C13—C14—C8	−68.8 (2)
C16—O8—C7—C8	27.7 (3)	C7—C8—C14—C15	61.3 (2)
C5—C6—C7—O8	−69.4 (2)	C20—C8—C14—C15	179.31 (17)
C5—C6—C7—C8	52.7 (3)	C9—C8—C14—C15	−62.8 (2)
O8—C7—C8—C20	−156.51 (17)	C7—C8—C14—C13	−166.73 (17)
C6—C7—C8—C20	86.2 (2)	C20—C8—C14—C13	−48.70 (19)
O8—C7—C8—C14	−49.6 (2)	C9—C8—C14—C13	69.2 (2)
C6—C7—C8—C14	−166.86 (19)	C1'—O10—C15—C16	−57.0 (2)
O8—C7—C8—C9	73.7 (2)	C1'—O10—C15—C14	69.3 (2)
C6—C7—C8—C9	−43.6 (2)	C13—C14—C15—O10	71.0 (3)
C7—C8—C9—C11	179.43 (18)	C8—C14—C15—O10	−170.89 (16)
C20—C8—C9—C11	49.1 (2)	C13—C14—C15—C16	−164.80 (18)
C14—C8—C9—C11	−59.0 (2)	C8—C14—C15—C16	−46.7 (2)
C7—C8—C9—C10	45.3 (2)	C7—O8—C16—O9	171.5 (2)
C20—C8—C9—C10	−85.0 (2)	C7—O8—C16—C15	−13.0 (3)
C14—C8—C9—C10	166.82 (17)	O10—C15—C16—O9	−35.3 (3)
C2—C1—C10—C19	−62.7 (2)	C14—C15—C16—O9	−162.7 (2)
C2—C1—C10—C5	56.3 (2)	O10—C15—C16—O8	149.45 (18)
C2—C1—C10—C9	170.74 (19)	C14—C15—C16—O8	22.0 (3)
C4—C5—C10—C1	−49.7 (2)	C13—O7—C20—C8	−6.9 (2)
C6—C5—C10—C1	179.60 (17)	C7—C8—C20—O7	148.59 (17)
C4—C5—C10—C19	67.7 (2)	C14—C8—C20—O7	35.0 (2)
C6—C5—C10—C19	−63.0 (2)	C9—C8—C20—O7	−81.7 (2)
C4—C5—C10—C9	−165.37 (18)	C22—O6—C21—O5	0.9 (4)
C6—C5—C10—C9	64.0 (2)	C22—O6—C21—C13	−175.2 (2)
C11—C9—C10—C1	57.6 (2)	O7—C13—C21—O5	−61.0 (3)
C8—C9—C10—C1	−170.06 (18)	C14—C13—C21—O5	−173.3 (2)
C11—C9—C10—C19	−64.1 (3)	C12—C13—C21—O5	54.7 (3)
C8—C9—C10—C19	68.3 (2)	O7—C13—C21—O6	115.2 (2)
C11—C9—C10—C5	172.60 (18)	C14—C13—C21—O6	2.8 (3)
C8—C9—C10—C5	−55.0 (2)	C12—C13—C21—O6	−129.1 (2)
C8—C9—C11—O3	−84.4 (2)	C15—O10—C1'—O11	−1.1 (3)
C10—C9—C11—O3	48.7 (2)	C15—O10—C1'—C2'	177.03 (19)
C8—C9—C11—C12	40.2 (2)	O11—C1'—C2'—C3'	−169.2 (3)
C10—C9—C11—C12	173.40 (18)	O10—C1'—C2'—C3'	12.7 (4)
O3—C11—C12—O4	−153.84 (17)	C1'—C2'—C3'—C4'	2.3 (5)
C9—C11—C12—O4	81.7 (2)	C1'—C2'—C3'—C5'	−179.0 (3)

### Hydrogen-bond geometry (Å, º)

**Table:**

*D*—H···*A*	*D*—H	H···*A*	*D*···*A*	*D*—H···*A*
O2—H2*A*···O1	0.82	2.17	2.629 (3)	116
O3—H3*A*···O11^i^	0.82	2.09	2.911 (2)	173
O4—H4*A*···O9^ii^	0.82	2.41	3.180 (2)	157
O4—H4*A*···O8^ii^	0.82	2.33	3.066 (2)	149
C11—H11*A*···O9^ii^	0.98	2.54	3.368 (4)	142
C5′—H5′*B*···O1^iii^	0.96	2.76	3.650 (3)	155

Symmetry codes: (i) −*x*, *y*+1/2, −*z*+1/2; (ii) −*x*+1, *y*+1/2, −*z*+1/2; (iii) −*x*+1/2, −*y*+1, *z*+1/2.
